# Frequency modulated continuous wave LiDAR with expanded field-of-view based on polarization-splitting metasurface

**DOI:** 10.1515/nanoph-2025-0183

**Published:** 2025-07-28

**Authors:** Kelan Chen, Jitao Ji, Xueyun Li, Zhiyuan Lin, Zhizhang Wang, Jiacheng Sun, Jian Li, Chunyu Huang, Pan Dai, Jitao Cao, Xiangfei Chen, Shining Zhu, Tao Li

**Affiliations:** National Laboratory of Solid State Microstructures, Key Laboratory of Intelligent Optical Sensing and Manipulation, Jiangsu Key Laboratory of Artificial Functional Materials, College of Engineering and Applied Sciences, C ollaborative Innovation Center of Advanced Microstructures, 12581 Nanjing University, Nanjing, 210093, China

**Keywords:** frequency-modulated continuous wave LiDAR, polarization beam-splitting metasurface, field of view expansion, dual-polarization state scanning

## Abstract

Frequency modulated continuous wave (FMCW) light detection and ranging (LiDAR) has recently become a research hotspot in the fields of autonomous driving and intelligent perception due to its high-precision ranging and velocity measurement capabilities. However, the existing LiDAR systems are usually challenged in expanding the field-of-view (FOV), which often comes at the expense of beam quality and degrades the detection accuracy and signal-to-noise ratio. On the other hand, the complexity of data processing algorithms may introduce significant measurement inaccuracies, potentially leading to substantial deviations in the final results. These two constraints limit the performance of LiDAR in complex scenarios. To address these issues, this paper proposes a new architecture for FMCW LiDAR that employs a geometric metasurface as a polarization splitter for expanded FOV of beam steering. With the combination of mechanical scanning mirror and metasurface, the scanning FOV has been successfully enlarged from 64° × 20° to 64° × 40°. Simultaneously, millimeter-level precision was achieved in distance measurement, along with an average relative error of 9 mm/s in velocity measurement, which confirms stable and precise system performance. This approach not only broadens the scanning range but also preserves the measurement accuracy of FMCW technology. This paper innovatively combines polarization beam-splitting metasurface with FMCW technology to achieve high-precision measurement over a wide field of view, providing a promising new technical pathway for the technological evolution of future LiDAR systems.

## 1 Introduction

LiDAR has emerged as a pivotal high-precision sensing technology, leveraging its high-resolution 3D imaging capabilities, excellent resistance to interference, and all-weather operability to achieve widespread adoption across domains such as autonomous driving, environmental monitoring, precise positioning, and mapping [1], [2], [3], [4], [5]. Traditional time-of-flight (ToF) LiDAR systems, despite their technological maturity and widespread adoption, still face several limitations, including susceptibility to ambient light interference, dependence of ranging accuracy on the pulse width of the laser, and the inability to directly measure target velocity, which requires multi-frame point cloud data analysis for estimation, thereby increasing computational load [6], [7], [8], [9]. In contrast, FMCW LiDAR systems not only achieve higher measurement accuracy compared to traditional TOF ones but also enable simultaneous distance and velocity detection through coherent beat frequency technology, significantly enhancing resistance to ambient light interference [10], [11], [12].

The scanning module, serving as the fundamental component in LiDAR-based 3D environmental perception systems, demonstrates dynamic coupling properties in its FOV that critically govern the integrity of target information retrieval [13], [14]. Current industrial-grade LiDAR systems primarily rely on macroscopic mechanical scanning to achieve 360° FOV coverage but are constrained by their large size, high cost, and short lifespan. Current scanning technologies face inherent limitations in achieving wide FOV coverage. Micro-electro-mechanical systems (MEMS) are typically constrained to FOVs below 25° × 15° [15], while optical phased arrays (OPAs) can reach 60° FOV but face significant manufacturing challenges [16], [17]. Liquid crystal modulators exhibit even narrower FOVs (<20°) [18], [19], and acousto-optic deflectors (AODs) are fundamentally limited to maximum 2° FOV [20], [21]. These inherent limitations in the FOV highlight the persistent challenge of developing scanning systems that can integrate wide-angle coverage with the requirements of practical implementation.

Recently, new breakthroughs in technology have been made based on metasurfaces, which provide powerful manipulation on light-field through subwavelength nanostructures in an ultra-thin framework [22], [23], [24], [25], [26]. Metasurfaces, having shown transformative applications in achromatic lenses [27], holographic imaging [28], and optical neural networks [29], would possibly provide a distinctive technological pathway for LiDAR systems. A study has been reported that through the integration of dielectric metasurfaces with liquid crystal modulation, the FOV of the spatial light modulator (SLM) was successfully enhanced to 22°, significantly surpassing the mere 0.7° FOV of conventional SLMs [30]. A research presents a high-efficiency metasurface-based optical beam scanning system with a complex quasi-three-dimensional subwavelength structure, which achieves a 144° × 144° FOV [31]. A team employed metasurfaces to expand the field of view of AOD from narrow 2° to 150°, enabling high-speed, wide-FOV TOF LiDAR [32]. While these works have significantly improved the FOV, their metasurface structures require complex fabrication processes, and the ToF-based structured light approach shows limited accuracy in dynamic scenarios.

In this work, we propose a novel FMCW LiDAR system. By using the phase gradient method, we design a polarization-splitting metasurface and integrate it with a scanning galvanometer to obtain dual-polarization beams, thereby significantly expanding the imaging FOV. This approach not only improves the imaging FOV performance but also maintains high-precision ranging and velocity measurement capabilities, paving the way for the development of the next-generation LiDAR technology.

## 2 Principle and design

**Figure 1:**
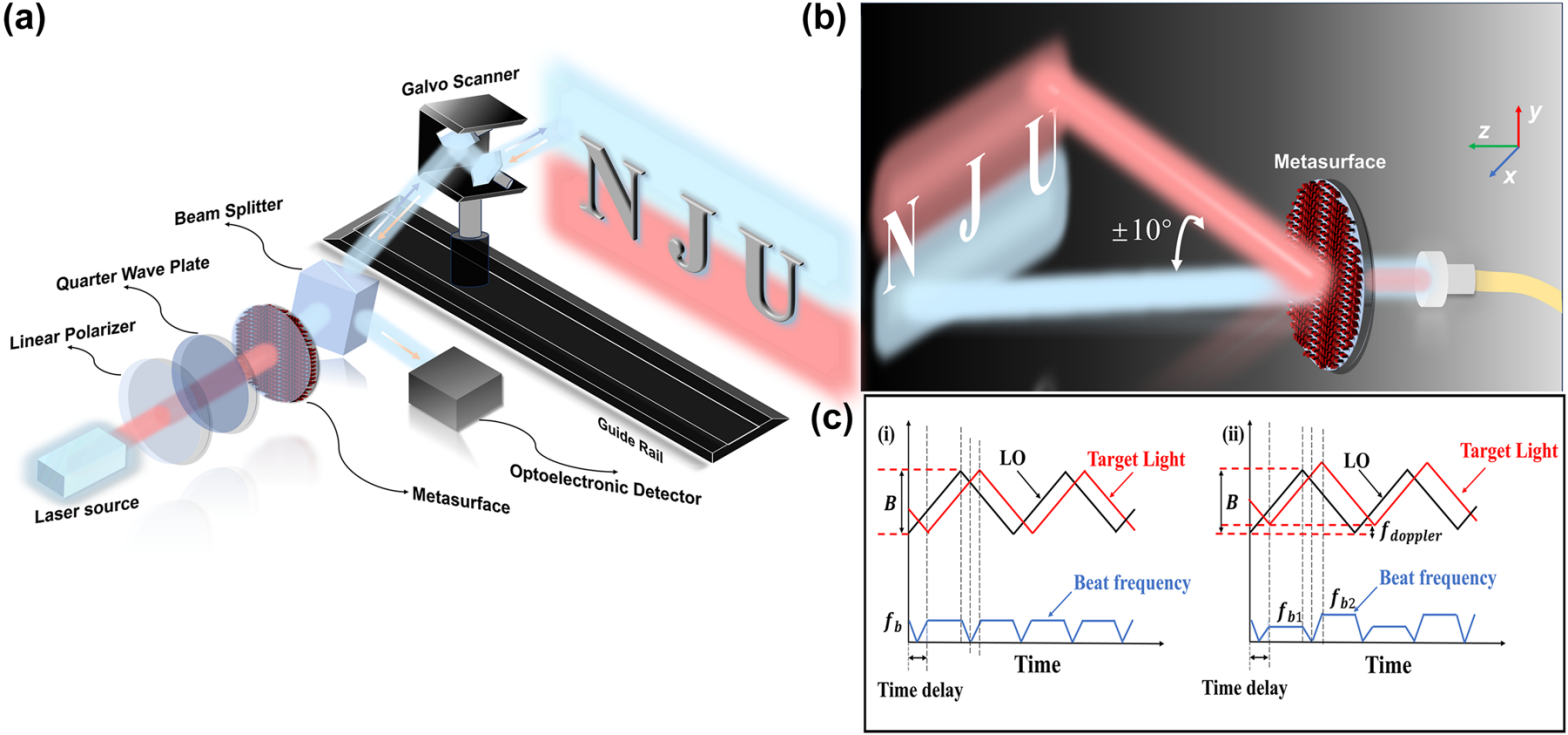
Schematic diagrams of a polarization beam-splitting metasurface-based FMCW LiDAR. (a) Spatial optical path design of metasurface-integrated LiDAR. (b) Metasurface-enabled functionalities for FMCW LiDAR. (c) Dual-mode detection principle of FMCW LiDAR. (i) Principle of ranging. (ii) Doppler velocimetry principle.

Figure 1a shows the scheme of the FMCW LiDAR system based on a polarization beam-splitting metasurface. The proposed system leverages the unique polarization beam-splitting properties of the metasurface to achieve an effective expansion of the scanning FOV. The specific working principle is described as follows. Firstly, the laser output is converted into linearly polarized light by using a linear polarizer. Then, the linearly polarized light is modulated into either left-handed polarization (LCP) or right-handed circular polarization (RCP) states via a quarter-wave plate. When these beams with different polarization pass through the metasurface, they undergo predefined angular deflections with angular deviations of ±10°, as shown in Figure 1b. The deflected beams are then directed by a beam splitter toward a two-dimensional galvanometer system, enabling target scanning. The two polarization states, after modulation by the galvanometer system, are respectively mapped to two distinct regions of the target scene. The blue field of view corresponds to LCP incidence, while the red field of view is associated with RCP incidence. The reflected echo signals from the target are received by the photodetector along the path through the beam splitter (BS). By synergistically combining polarization state modulation with metasurface-induced deflection, and integrating a rail displacement mechanism, the system achieves panoramic imaging over a significantly expanded FOV. The complete FMCW system and specific components used see Section 1 of the Supplementary materials. Different from traditional ToF LiDAR, which obtains the distance of target objects by measuring time, the proposed method uses infrared light with a frequency that changes linearly with time for direct coherent detection. The intrinsic light emitted by the source and the light scattered back from the object are subjected to beat-frequency mixing. As illustrated in Figure 1c(i) by the FMCW ranging principle, the distance *L* between the laser source that generates the modulated signal and the target can be written as

(1) $$L=\frac{f_{b}cT}{2B}$$

where *f*_*b*_ is the measured beat-frequency, *c* is the speed of light in a vacuum, and *T* is the modulation period of the triangular wave signal. The FMCW scheme is demonstrated to have a significant advantage over ToF method, as it enables the simultaneous measurement of a distance and velocity. For a moving object, a Doppler frequency shift *f*_Doppler_ will be introduced during the measurement process due to the relative motion between the object and the observer as shown in Figure 1c(ii). Two distinct beat frequency signals are generated during the test, and the relationship between these two signals is expressed in equation (2), corresponding to the frequency modulation induced by both the object’s motion and system’s inherent frequency sweep. From this, the distance and velocity information of moving object can be directly obtained, as shown in equation (3).

(2) $$\left\{\begin{array}{c} f_{b1}=f_{b}-f_{\text{doppler}}\;\\ f_{b2}=f_{b}+f_{\text{doppler}}\; \end{array}\right.$$

(3) $$\left\{\begin{array}{c} v_{r}=\frac{f_{\text{doppler}}\cdot \lambda }{2}=\frac{\left(f_{b2}-f_{b1}\right)\cdot \lambda }{4}\;\\ L=\frac{\left(f_{b2}+f_{b1}\right)\cdot c}{2\gamma }\; \end{array}\right.$$

Here, the central wavelength *λ* of the DFB laser used for frequency sweeping is 1,545.6 nm. The frequency modulation slope *γ* is defined as the ratio of the actual modulation bandwidth to half of the tuning period. By calculating the sum and difference of two peaks and combining the actual tuning slope obtained after performing nonlinear correction on the DFB laser in the experiment, the distance and velocity of a moving object can be accurately determined. The frequency sweep was modulated at 10 kHz, with the wavelength tuning range or the modulation bandwidth, approximately 12 GHz which is mentioned at Section 2 of the Supplementary materials.

The polarization beam splitting metasurface was fabricated using silicon dioxide (SiO_2_) as the substrate material, with crystalline silicon (Si) employed for the nanopillar structures, according to the operational requirements of the wavelength used in the experimental setup. Based on the Pancharatnam–Berry phase, a phase-gradient metasurface was designed to impart a 10° deflection angle to the incident light, enabling this polarization-division detection scheme. The refracted light follows the generalized Snell’s law as following equation (4):

(4) $$n_{t}\sin\theta_{t}-n_{i}\sin\theta_{i}=\frac{\lambda }{2\pi }\frac{d\Phi }{dx}=\frac{\lambda }{\pi }\frac{d\varphi \left(x\right)}{dx}$$

Where *n*_*i*_ and *n*_*t*_ are the refractive indices of the medium before and after traversing the metasurface. *θ*_*i*_ and *θ*_*t*_ are the incident and refraction angles, respectively. $\frac{d\Phi }{dx}$ represents the phase gradient of the metasurface. According to the required deflection angle, the phase distribution of the metasurface is as following equation (5):

(5) $$\Phi \;=\;\frac{2\pi }{\lambda }\sin\left(\theta \right)\cdot x,\theta =10^{◦}$$

**Figure 2:**
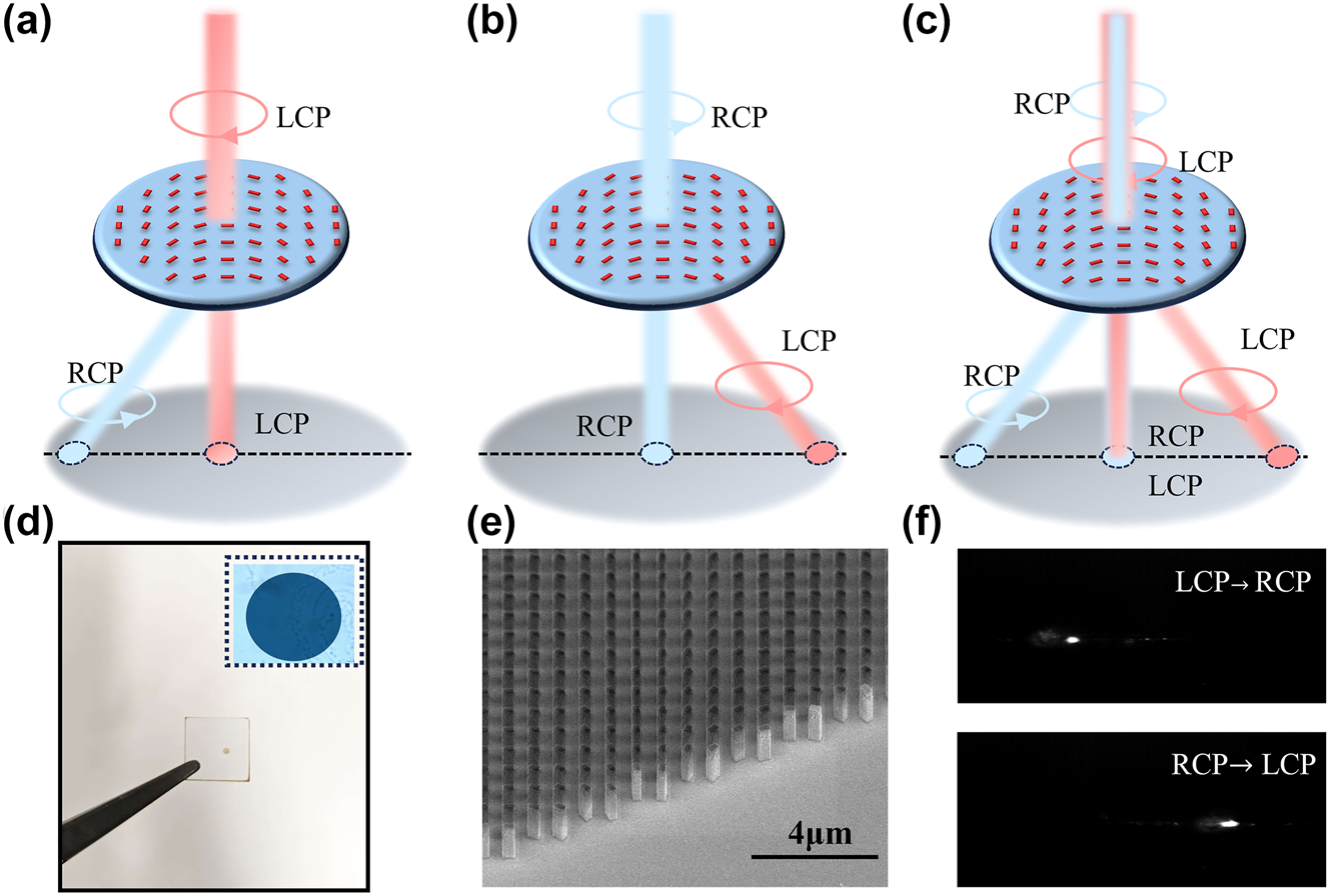
Design principles and experimental characterization of polarization beam splitter based on metasurface. (a–c) Working principle of polarization state conversion. (d) Optical image of the polarization beam-splitting metasurface sample, with a 1-mm-diameter silicon-based metasurface optical micrograph under 10× objective lens shown in the upper right inset. (e) Electron microscopy characterization of the metasurface. (f) Far-field imaging of polarization conversion.

The polarization conversion principle of the proposed metasurface is illustrated in Figure 2a–c; when LCP is incident on the metasurface, it is converted into RCP and deflected to the left at 10°, while the unconverted light continues to transmit straight through. Conversely, when RCP is incident, it is converted into LCP and deflected in the opposite direction at the required angle. For mixed-polarization incident light, the output combines the behaviors of both LCP and RCP incident, resulting in a superposition of the effects seen in Figure 2a and b. Using finite-difference time-domain (FDTD) simulations and particle swarm optimization, we determined the optimal structural parameters for the nanopillars: a length of 361.2 nm, width of 242.96 nm, and height of 1,200 nm. This configuration achieves a transmittance of 94 % and a polarization conversion efficiency of 99 %. The simulation of the entire metasurface shows that the transmittance and polarization conversion efficiency are 82 % and 97 %, respectively. Based on these parameters, a circular metasurface with a diameter of 1 mm was fabricated on a 2 mm-thick silicon wafer as shown in Figure 2d, where the upper-right corner shows a magnified view of the metasurface under a 10× microscope objective. Before integrating the metasurface into the full system, we qualitatively tested its polarization conversion performance, which is shown in Section 3 of the Supplementary materials. The polarization conversion efficiency and transmittance of the metasurface were measured to be 72.5 % and 60.8 %, respectively, which shows certain discrepancies from the theoretical simulation that may be attributed to calibration test deviations and processing errors Section 2 of the Supplementary materials. A representative scanning electron microscopy (SEM) image of the fabricated metasurface’s boundary region is presented in Figure 2f. Well-defined nanostructures with smooth sidewall profiles are observed, and no structural deformation or collapse is detected. These morphological characteristics are confirmed to satisfy the fabrication fidelity requirements for consistent optical performance. The far-field intensity profile of the transmitted beam through the metasurface was captured by an infrared CCD camera in Figure 2e. Systematic experimental measurements confirmed that deflection angles of the converted polarization states showed excellent agreement with theoretical predictions.

## 3 Results

**Figure 3:**
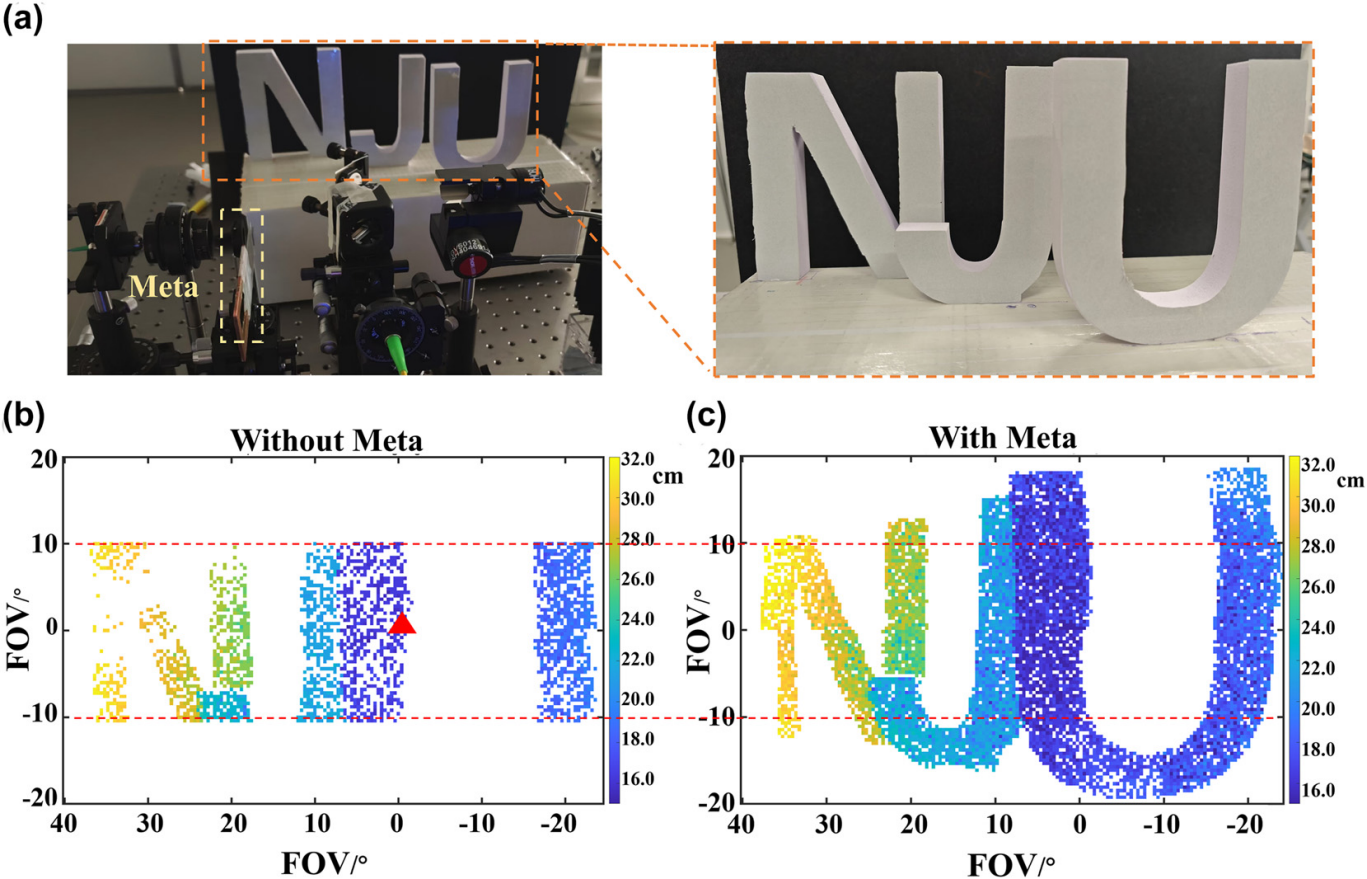
FMCW LiDAR scanning FOV of imaging based on polarization-splitting metasurface. (a) Experimental imaging scene “NJU,” where three letters are arranged in a staggered configuration. (b) Scanning results without metasurface polarization multiplexing, showing the FOV of 64° × 20°. (c) Stitching result of LCP and RCP states by scanning in both polarization states, where the total imaging field of view is expanded to 64° × 40°, doubles the case without metasurface.

The experimental imaging setup comprised three polyvinyl chloride (PVC) letter structures forming the acronym “NJU,” arranged with a specific spatial configuration. To mitigate background light interference, a black plastic substrate was positioned behind the target objects. This configuration is schematically illustrated in Figure 3a. The scanning system employs a Thorlabs GVS102 two-axis galvanometer scanner in 0.5 V/° mode with a step size of 0.1 V. For FOV imaging with different polarization states, a unified galvanometer scanning range is employed, with a bipolar driving voltage ranging from −6 V to +10 V applied to the horizontal axis and a symmetric voltage sweep maintained between −2.5 V and +2.5 V on the vertical axis. This corresponds to a horizontal FOV of −24° to 40° and a vertical FOV of −10° to +10°, where the positive/negative definition of the voltage directly aligns with the sign convention of the angles. To calibrate the system stability before testing, the Allan deviation of the system was measured, as shown in Section 5 of the Supplementary materials. The ranging image acquired through direct laser scanning without metasurface modulation using the specified galvanometer parameters is shown in Figure 3b. In this image, the outlines of the three-letter NJU pattern cannot be clearly identified. Different color values represent the actual distance between the galvanometer and the object, while color gradients reflect subtle changes in the relative position. The red triangle in the figure represents the reference point for the FOV of the light that has not been modulated by the metasurface when the galvanometer voltages are all at 0 V. To facilitate experimental convenience, the metasurface was configured for horizontal beam splitting, followed by a two-mirror galvanometer system to convert it into vertical beam splitting, effectively expanding the vertical field of view. Figure 3c demonstrates the combined FOV from scanning with both LCP and RCP, where the stitched field of view measuring 64° horizontally by 40° vertically successfully reconstructs the complete three-dimensional morphology of the NJU letter pattern while accurately characterizing their spatial relationships, with errors measured to be on the millimeter scale. By the way, the FOV switching is achieved by adjusting the galvanometer to center the beam after polarization state conversion, coupled with slight adjustments to the BS position and horizontal translation of the galvanometer along a slide rail. During polarization state conversion, a rigid breadboard is used to ensure the synchronous displacement of the galvanometer and the object, thus maintaining the consistency of the imaging FOV. By integrating the polarization-splitting metasurface, it overcomes the mechanical scanning limits of galvanometers, doubling the effective FOV. The metasurface designed in this system provides a limited deflection angle of 10°, which is intended to verify the feasibility of the beam-splitting scheme for field-of-view expansion. Large-angle deflection can be easily achieved using the existing design method, and greater angular expansion can be realized through collaboration with the galvanometer.

**Figure 4:**
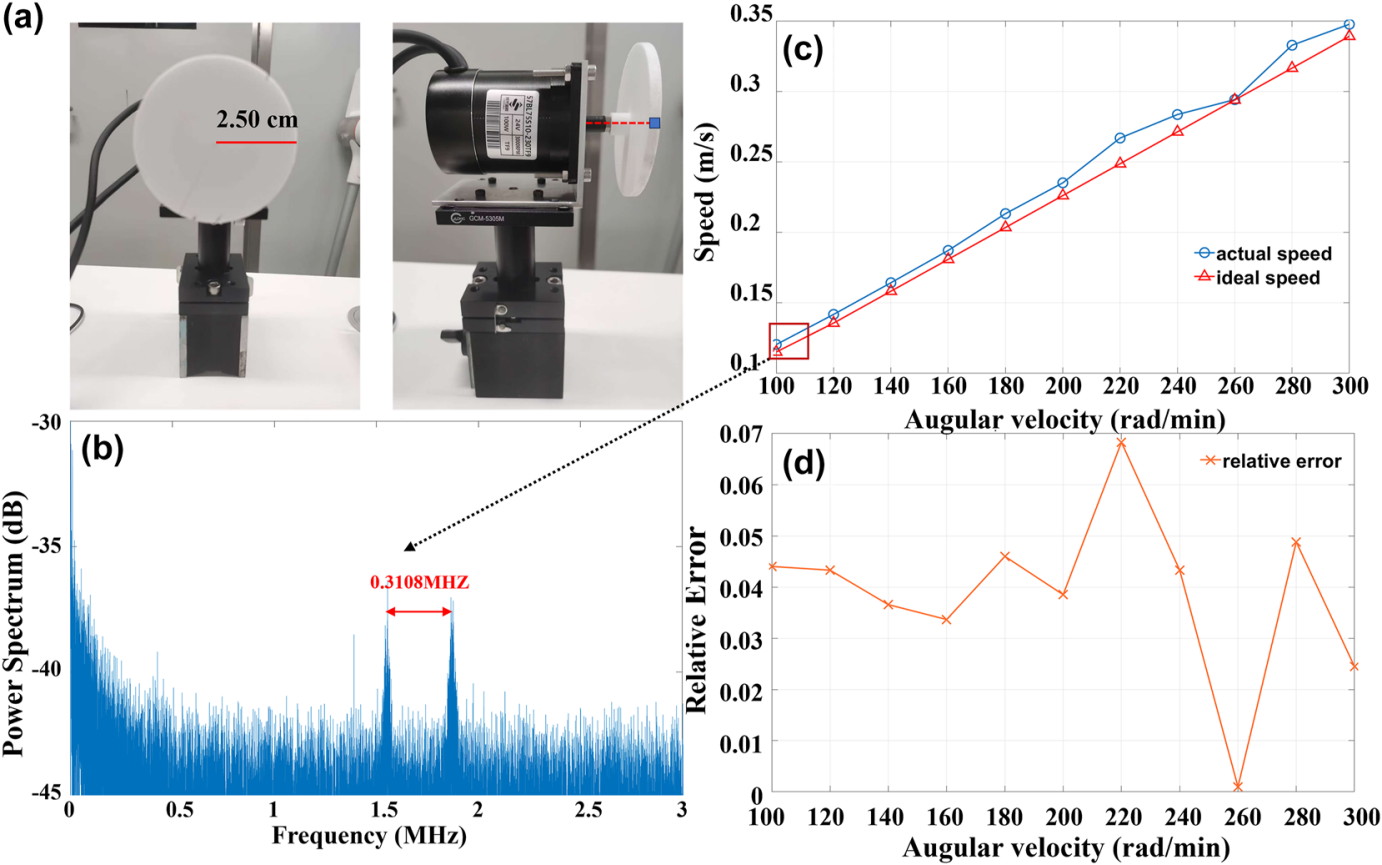
FMCW LiDAR velocimetry system setup and results. (a) Speed measurement setup: Electric motor with a white rotating disk. (b) Experimental velocity FFT spectrum: Two correlated peaks induced by Doppler shift. (c) Measured velocity values at different disk rotation speeds. (d) Relative error between theoretical and actual disk speeds.

The velocimetry setup integrated into the proposed LiDAR system is demonstrated in Figure 4a. The velocimetry device is composed of an electric motor and a white rotating disk with a radius of 2.50 cm. The motor driving unit is controlled through computer serial communication to adjust the disk rotation speed. In the experiment, the laser beam can irradiate any region above or below the blue dot on the extension line of the rotation axis to measure the radial velocity of the disk. It was incident on the disk at sinα = 9/25 for radial, where the velocity is given by the following equation (6):

(6) $$\upsilon_{r}=\omega r\sin\alpha$$

where *ω* is the rotational speed, *r *is the radius of the disk, and α is the angle between any point and the blue dot in the Figure 4a.

During disk rotation at 100 rad/min, the beat frequency is split into two distinct peaks with a frequency difference of 0.3108 MHz due to the Doppler shift effect, as illustrated in Figure 4b. These peaks are processed to derive both velocity and distance information of the rotating disk. Experimental measurements were performed at uniformly distributed rotation speeds ranging from 100 to 300 rad/min, with a step size of 20 rad/min corresponding to a velocity interval of 2 cm/s for velocity testing. As shown in Figure 4c, the blue velocity curve demonstrated near-ideal linear congruence with the theoretical velocity distribution of the disk at the rotational speed marked in red, validating the reliability and accuracy of the results. The experiment can test velocities in the cm/s range, and the mean deviation between the experimental and theoretical velocities is only 9 mm/s. Further, the mean relative velocity error remains below 4 % when operating at velocity interval of 2 cm/s as presented in Figure 4d.

Considering systematic errors, testing errors, etc., this discrepancy is considered reasonably acceptable given calibration inaccuracies and experimental limitations. Verifying the system has practical applicability in speed measurement.

## 4 Discussion and conclusion

In conclusion, the article innovatively develops an FMCW LiDAR system based on polarization-splitting metasurface. The system ensures measurement accuracy through an FMCW coherent detection architecture. Meanwhile, the polarization-splitting metasurface splits the beam into dual polarization states for FOV stitching, achieving a twofold FOV expansion compared to conventional galvanometer systems. This solution provides new insights for the development of integrated high-precision LiDAR systems, demonstrating significant application value in autonomous driving and industrial inspection fields. Combining the advantages of wide FOV and high precision measurement, the system pioneers new directions for next-generation LiDAR technology.

However, there is still room for further improvement in future research. First, in terms of hardware implementation, the performance of system is constrained by data acquisition and processing capabilities. In subsequent developments, the imaging rate can be further enhanced by adopting high-speed acquisition cards and FPGA/ASIC chips. Second, customizable imaging of target areas can be achieved through the collaborative coordination between metasurface deflection angles and galvanometer scanning angles, as well as the setting of galvanometer scanning precision. Additionally, it is expected that the volume of the metasurface-based FMCW LiDAR can be further reduced to achieve system miniaturization through on-chip integrated optical phased arrays and the future integration scheme in Section 4 of the Supplementary materials. The experimental architecture proposed in this paper provides a novel solution for future large FOV and high-precision LiDAR systems.

## Supplementary Material

Supplementary Material Details
