# Supplementary material for: Frequency modulated continuous wave LiDAR with expanded field-of-view based on polarization-splitting metasurface
Source: Nanophotonics. 2025 Jul 28;14(17):2901–8. doi: 10.1515/nanoph-2025-0183 (PMC12397743; doi:10.1515/nanoph-2025-0183)
Supplement: Supplementary file 1 — Supplementary Material Details [file j_nanoph-2025-0183_suppl_001.docx]

**Supporting information for**

**Frequency modulated continuous wave LiDAR with expanded field-of-view based on polarization-splitting metasurface**

Kelan Chen^a^, Jitao Ji^a^, Xueyun Li^a^, Zhizhang Wang^a^, Jiacheng Sun^a^, Jian Li^a^, Chunyu Huang^a^, Pan Dai^a^, Jitao Cao^a^, Xiangfei Chen^a^, Shining Zhu^a^, and Tao Li^a, *^

^a^ National Laboratory of Solid State Microstructures, Key Laboratory of Intelligent Optical Sensing and Manipulation, Jiangsu Key Laboratory of Artificial Functional Materials, College of Engineering and Applied Sciences, Nanjing University, Nanjing, China, 210093.

^*^*e-mail:* [*taoli@nju.edu.cn*](mailto:taoli@nju.edu.cn)

**Section Ⅰ: FMCW LiDAR with metasurface**


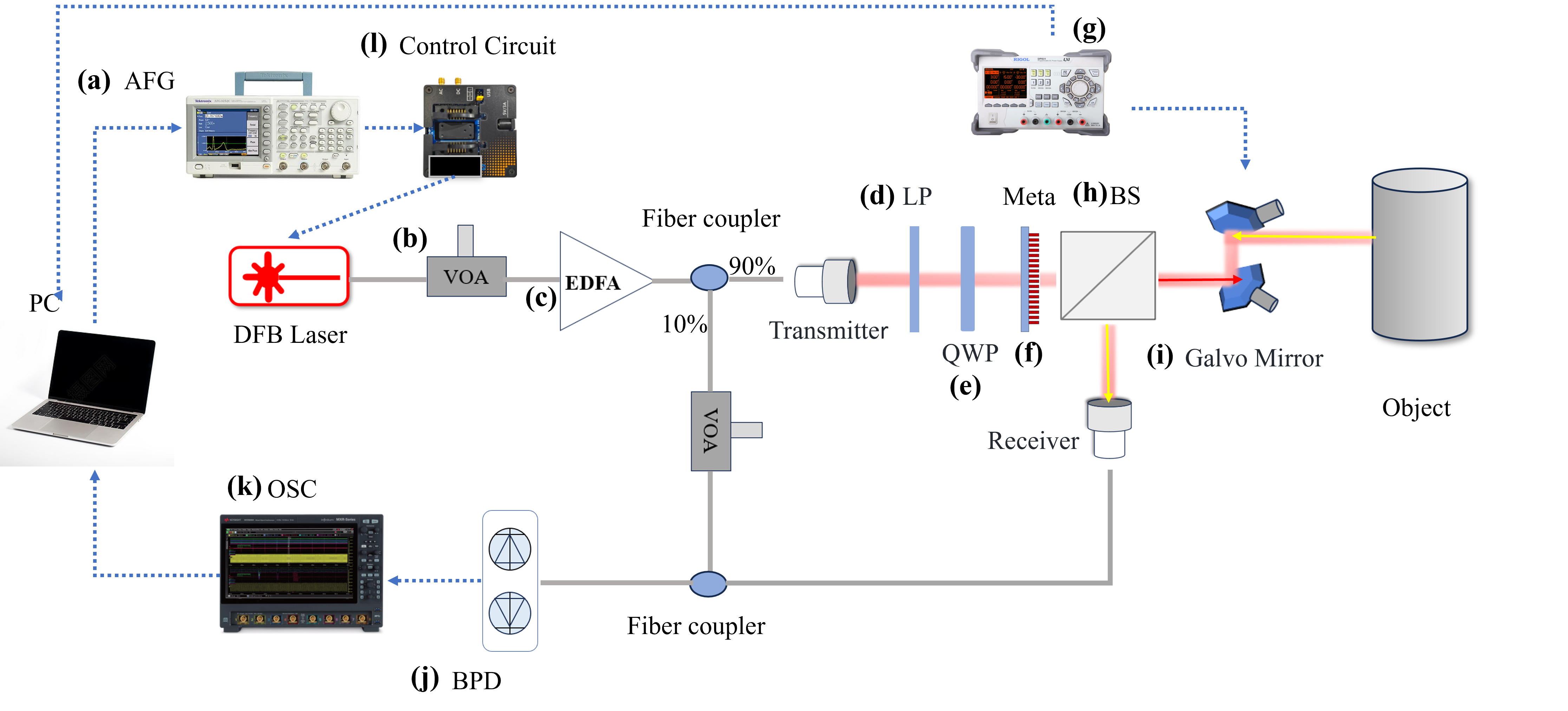


**Figure 1:** Schematic diagram of FMCW LIDAR system with field-of-view expanded based on polarization-splitting metasurface. (a) Arbitrary function generator. (b) Variable attenuator. (c) Erbium doped fiber amplifier. (d) Linear polarizer. (e) Quarter wave plate. (f) Polarization-splitting metasurface. (g) Three-channel programmable voltage source. (h) Beam splitter. (i) Galvanometer mirror. (j) Balanced photodetector. (k) Oscilloscope. (l) Control circuit.

The proposed polarization-splitting metasurface-based FMCW LiDAR system as shown in figure 1, which comprises three principal modules: frequency-swept light generation, spatial measurement, and signal processing. In the frequency-swept light generation section, a linear optical frequency-modulated signal is produced by DFB laser whose central wavelength is 1545.6 nm driven by an arbitrary function generator (AFG). The AFG driving voltages are determined via computer-optimized nonlinear correction, where iterative calibration refines the modulation waveform to compensate for system nonlinearities. To ensure stable operation, the DFB laser's temperature and modulation current are regulated by a dedicated control circuit. The modulated optical signal undergoes power regulation through a VOA to achieve the optimal input power level (~0 dBm) for EDFA operation, followed by gain amplification via the EDFA to offset system optical losses, thereby guaranteeing adequate signal-to-noise ratio at the receiver. The amplified optical beam is divided by a 10:90 fiber-optic coupler, allocating 10% of the power as the local oscillator (LO) reference arm while directing 90% to the probe arm for target illumination. Before entering the scanning module, the beam's initial mixed polarization state is first collimated to linear polarization through a high-extinction-ratio linear polarizer (LP). Subsequently, a precision-mounted quarter-wave plate (QWP) is dynamically tuned to alternately generate left-circular polarization (LCP) and right-circular polarization (RCP) states. The spatial measurement module incorporates a polarization-splitting metasurface (f) that simultaneously performs angular beam deflection and polarization conversion, demonstrating a high deflection efficiency at the designated operating wavelength. The deflected beam is then directed to a high-performance galvanometer scanning system (h), where dynamic beam steering with a ±40° field of view (FOV) is achieved through precision voltage control via a ±10 V signal from a programmable three-channel source (g). The backscattered light from the target is collected and combined with the reference LO in a 2×2 fiber coupler. The resulting interference signal is detected by a balanced photodetector (j) and digitized for signal processing in computer. A fast Fourier transform (FFT) is performed on the acquired time-domain signal using an oscilloscope, and the peak of frequency in the power spectrum is extracted for distance and velocity calculations via post-processing algorithms. By scanning different parts of an object with a galvanometer mirror and processing the test data, three-dimensional imaging of the object can be achieved.

**Section Ⅱ:** **FMCW laser source nonlinearity calibration**

**
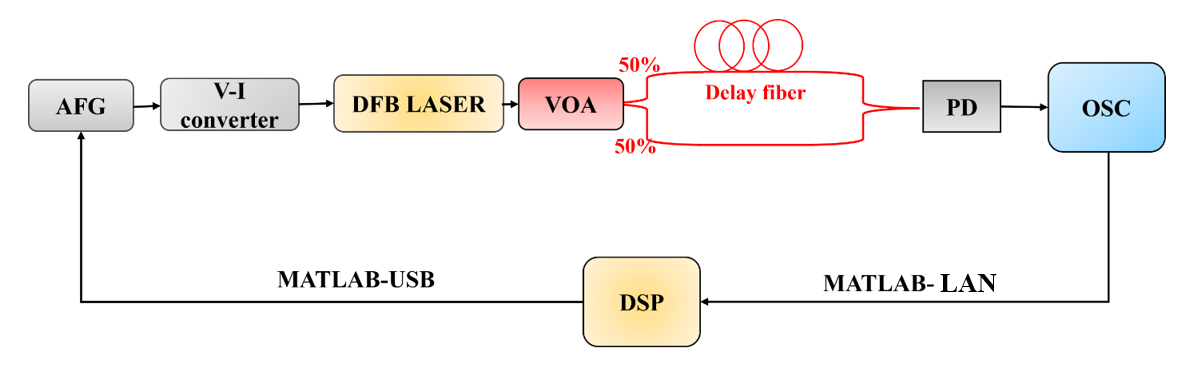
**

**Figure 2:** Open-loop automatic iterative calibration for FMCW Laser source nonlinearity

In the experimental setup, the externally modulated DFB laser exhibits nonlinear frequency chirping, which broadens the beat-note spectrum and degrades the ranging resolution of the FMCW LiDAR system. To address this issue, we implemented an iterative calibration framework to linearize the optical frequency sweep. As illustrated in Figure 2, the system configuration comprises a signal generator applying a triangular waveform to modulate the DFB laser, a delay fiber simulating target distances, and a digital signal processing unit for data analysis. We introduce a voltage-frequency error mapping algorithm that attributes frequency deviations from the ideal linear sweep to imperfections in the modulation waveform. The nonlinearity is defined as the ratio of the root-mean-square (RMS) optical frequency difference between the ideal and actual values to the modulation bandwidth. Leveraging MATLAB instrument control toolbox, we automated the calibration process via Ethernet and USB interfaces, enabling real-time data acquisition and voltage waveform generation.

Figure 3(a-b) compares the optical frequency profiles before and after calibration, demonstrating significant linearization across a 12 GHz modulation bandwidth at the signal modulation frequency of 10kHz. Spectral analysis in Figure 3(c-d) reveals pre-calibration nonlinearity values of 1.08% (up-chirp) and 2.06% (down-chirp), resulting in broadened spectra unsuitable for high-precision ranging. Post-calibration results in Figure 3(e-f) show dramatic improvements, with nonlinearity reduced to 0.0024% and 0.0016%, corresponding to enhancement factors of 491.7× and 1287.5×, respectively. These results validate the efficacy of our calibration method for practical FMCW applications requiring sub-millimeter precision.


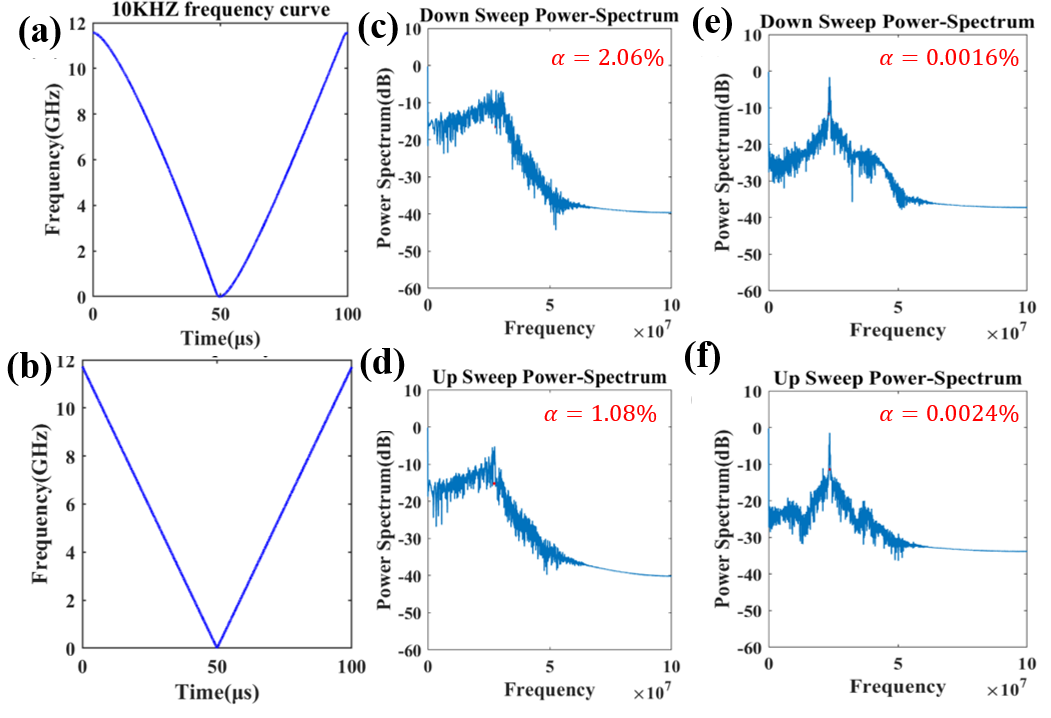


**Figure 3:** Nonlinearity calibration results. (a-b) Optical frequency curves before and after calibration; (c-d) Test spectraum of up-chirp and down-chirp before calibration; (e-f). Test spectra of up-chirp and down-chirp after calibration

**Section Ⅲ: Metasurface parameter characterization**


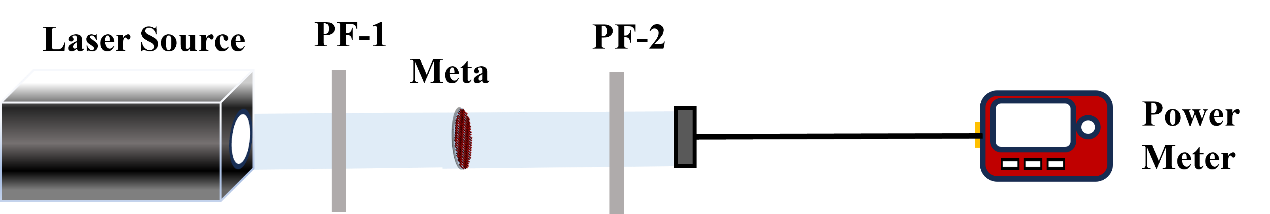


**Figure 4: T**he experiment of polarization conversion efficiency for metasurface

An experimental optical path was set up to test the polarization conversion efficiency and transmittance of the metasurface as shown in figure.4 and figure.5. The experimental design of polarization conversion efficiency for metasurface is as follows: The laser first passes through a polarizer (PF-1 in the figure) composed of a linear polarizer and a quarter-wave plate to generate a LCP or RCP beam. After the laser is incident on the metasurface, a majority of light (~72.5%) is converted to its orthogonal polarization state, with about 27.5% remains in the original polarization state. We place another polarizer, adjusted to the orthogonal polarization state, behind the metasurface to filter out the original one. The polarization conversion efficiency is calculated by using a power meter to measure the ratio of optical power with and without the polarizer filter (the second PF-2).


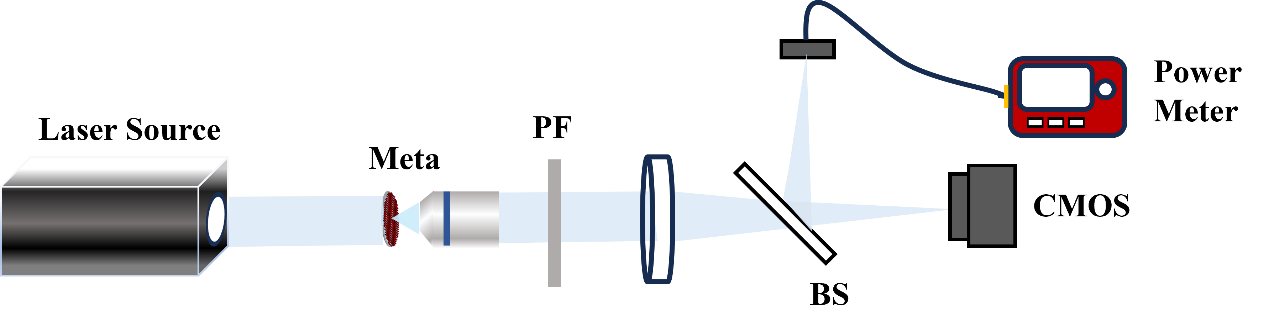


**Figure 5:** The experiment of transmittance efficiency for metasurface.

When measuring the transmittance, an objective lens adapter is used to focus light through a lens to obtain the far-field image of the metasurface light, and a CCD is employed to detect its intensity. By controlling the distances between the BS and the CCD, as well as between the BS and the power meter, to be identical, it ensures that measurements are taken on an equi-phase surface. The transmittance of the metasurface is obtained through the ratio of the powers of the two beams. In the experiment, the polarization conversion efficiency and transmittance of the metasurface were measured to be 72.5% and 60.8% respectively, which show certain discrepancies from the theoretical simulation design. The possible reasons may be attributed to calibration test deviations and processing errors.

**Section Ⅳ: Scheme of FOV switching**

In the experiment of this paper, it is indeed necessary to move the beam splitter and galvanometer to switch imaging scenarios during distance measurement. It appears to be complicated and less compactable for compact application. However, I should mention that in this work we are aiming to propose a scheme of combination of metasurface and FMCW technology as a proof-of-concept. At present stage, limited by the current experimental conditions, we used the traditional displacement stage and beam splitter (BS) used are relatively large in volume, which leads to a large distance from the metasurface to the galvanometer (~13 cm). Therefore, the designed ±10° deflection angle with metasurface corresponds to a distance of about 5.4 cm, which cannot be covered by the size of the galvanometer (2 cm × 2 cm). Then, we utilize a complicated movable system to show the working principle (see Fig.6).

For the practical application, this schemed will be further simplified by integrating the metasurface, polarization beam splitter and galvanometer to a compact form, and the metasurface-to-galvanometer distance will be reduced to smaller than 4 cm. Then the split beams will be covered by galvanometer, and there is no need to move the beam splitter and galvanometer (see Fig.7). In addition, the future integrated scheme can also reduce light propagation loss and improve energy utilization efficiency and practical application efficiency.


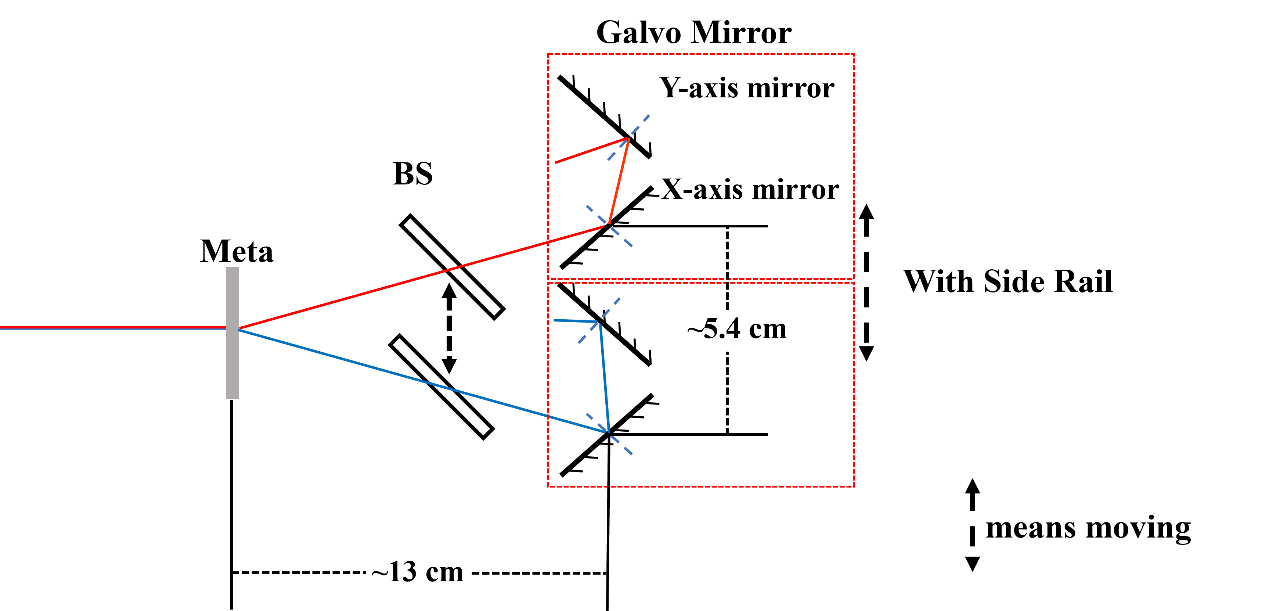


Figure 6. Optical path diagram of the current scheme for proof-of-concept demonstration


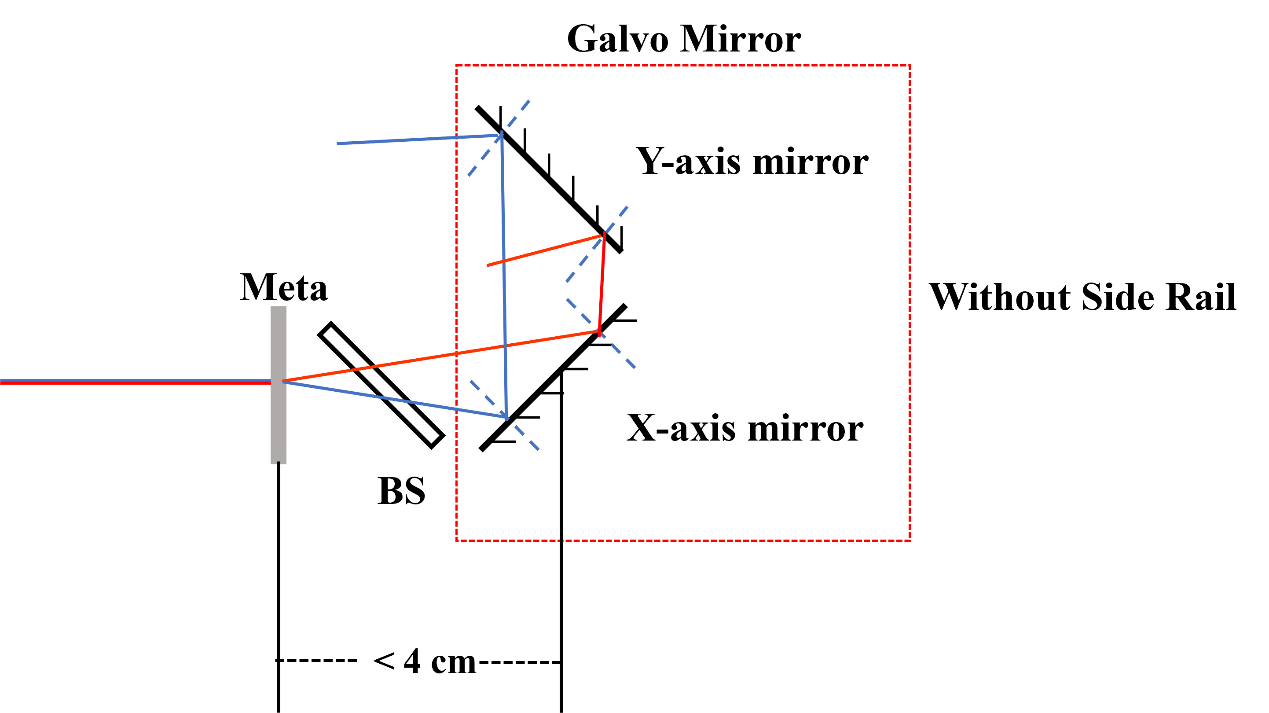


Figure 7. Optical path diagram of the future integration scheme

**Section Ⅴ: Allan deviation**


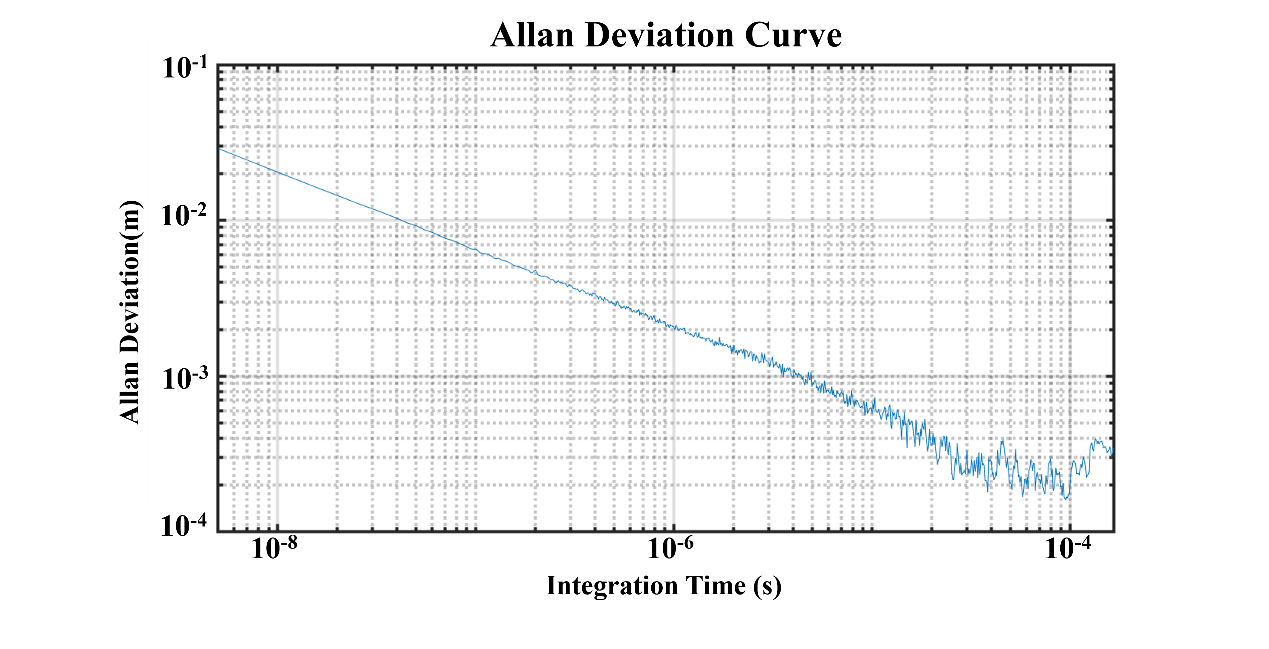


Figure 8. Allan deviation of the distance measurement versus intergration time.

To verify the stability of the galvanometer system, we used a sampling frequency of 200 MHz and collected 100,000 data samples. The analysis results show that under nanosecond-scale integration time, the allan deviation is at the centimeter level and continuously decreases as the integration time extends, reflecting the noise suppression effect. Due to the limitations of using an oscilloscope for data acquisition, the integration time is restricted. In future integrated solutions, hardware optimization (such as adopting high-speed acquisition cards) can be used to calculate the allan deviation over longer integration times, thereby characterizing the system stability more accurately.
